# Thromboelastography Reference Values for Third-Trimester Healthy Obstetric Patients in Northern Mexico

**DOI:** 10.1155/anrp/8871619

**Published:** 2025-03-06

**Authors:** S. Alvarado-Ramos, M. R. López-Gutiérrez, R. D. Nuñez-Alvar

**Affiliations:** ^1^Medical Center of High Specialty Gynecology and Obstetrics No. 23, Mexican Social Security Institute, Monterrey, Nuevo León, Mexico; ^2^School of Medicine and Health Sciences, Technological Institute and Higher Studies of Monterrey, Monterrey, Nuevo León, Mexico

**Keywords:** coagulation, goal-directed therapy, obstetric hemorrhage, reference values, thromboelastography, viscoelastic analysis

## Abstract

**Objective:** This prospective, descriptive, cross-sectional study aimed to establish kaolin-based thromboelastography reference values for previously known healthy third-trimester pregnancy patients.

**Methods:** The study included 280 patients aged 18–38 years who were admitted to labor or scheduled for elective c-sections. Blood specimens collected via IV catheters were immediately mixed with reagents, placed in coagulation cups, and subjected to 60 min of testing at 37°C using a Haemonetics TEG 5000 system. The Hoffman regression method calculated the reference values; furthermore, effect size determination was done using Cohen's *δ* for comparison of data from other sources.

**Results:** Patients had a median age of 26 (IQR 22–31), and their thromboelastography profile exhibited reference values for: *R* time (1–7 min), clot kinetics (1-2), angle (59°–82°), maximum amplitude (60–86 mm), and clot lysis at both 30 min (0%–6%) and 60 min (0%–8%). Results revealed significant differences in various thromboelastography parameters when comparing local patient cohorts against published reports, mainly European and North American counterparts. Shorter reaction times, enhanced clot kinetics, larger angles, and higher maximum amplitude, curve amplitude at 30 min, and amplitude at 60 min indicated distinct coagulation profiles and behaviors in the northeastern region of Mexico.

**Conclusion:** Reference values for the Northern region of Mexico have been calculated and are characterized by a shorter clot reaction time, faster clot dynamics, higher angle values, overall greater curve amplitude, and no differences in enzymatic lysis activity compared to samples from other geographic regions.

## 1. Introduction

Obstetric hemorrhage still represents a significant risk for maternal morbidity and mortality, especially in clinical settings where hemostatic alterations coincide with underlying obstetric pathologies, including but not limited to major blood loss, placenta accreta/percreta, antiplatelet therapy, and patients with inherited coagulopathies [1]. Managing major bleeding events requires clinicians to immediately interpret the patient's coagulation tests and effectively adjust the patient's coagulation profile, thereby preventing further deterioration and promoting optimal health outcomes [2]. The application of viscoelastic goal-directed therapy (GDT) has proven to be highly effective in managing patients with obstetric hemorrhage. The appropriate use of the technology, proficient interpretation, and subsequent transfusion planning have decreased mortality, perioperative morbidity, total transfusion, and waste of blood products [3, 4]. The reaction time (R-Time), clot dynamics, angle, and maximum amplitude [R, K, *α*, MA] provide the most valuable information about the blood coagulation profile, enzymatic activity, clot kinetics, and mechanical features [5, 6]. The test can generate clot parameters within 10–20 min. This feature enables the rapid development of a tailored transfusion strategy for the patient. It improves the blood product approach throughout the patient's clinical evolution in the ever-changing OR scene [7–9].

To effectively distinguish between healthy hemostatic states and altered ones using thromboelastography (TEG)-generated targets, one must establish reference intervals carefully, tailored to specific demographics. Doing so ensures accurate discrimination while minimizing the risk of misinterpretation or inappropriate responses [10].

It is well documented that pregnant women have an increased tendency toward hypercoagulability, characterized by increased activity of their coagulation factors, platelets, and fibrinogen levels [11]; moreover, they feature decreased endogenous anticoagulants and an increase in antifibrinolytic profile because of increments in PAI-2 [12]. However, we have not adequately characterized the parameters of patients in their third trimester until now.

Our center is a tertiary-level hospital providing gynecological, obstetrical, and neonatal medical services in seven Northern Mexican states. In 2021, the hospital attended 14,833 live births, 633 (3.89%) postpartum hemorrhage cases, and 68 (0.7%) massive bleeding events (defined as loss of > 50% blood volume). Since 2014, every hemorrhage case has received multidisciplinary care from rapid response teams, which has improved patient survival rates, decreased morbidity outcomes, and reduced the necessity for intensive therapy and hospitalization.

The management philosophy for these patients emphasizes GDT, along with prompt and well-informed clinical decisions intended to anticipate, control, and follow up on patients who experience obstetric hemorrhage. At our center, patients experiencing significant bleeding because of obstetric hemorrhage undergo transfusion GDT for optimal management. The established targets guiding this approach include the following: hemoglobin concentration: A target range of 70–80 g per liter (g/L) is maintained with red blood cells [13, 14]. Coagulation parameters: prothrombin time (PT) to achieve a time < 16 s or an international normalized ratio (INR) value below 1.5 with fresh frozen plasma (FFP) 10 mL/Ikg [9, 13]. Fibrinogen replacement therapy is utilized based on readiness, either by human fibrinogen concentrate or cryoprecipitate. The target fibrinogen level concentration of ≥ 2 g/L is aimed for during the acute phase of hemorrhage [15]. Target platelet count: During the acute process, levels between 50,000 and 75,000 platelets/μL with platelet apheresis [16].

**Figure 1:**
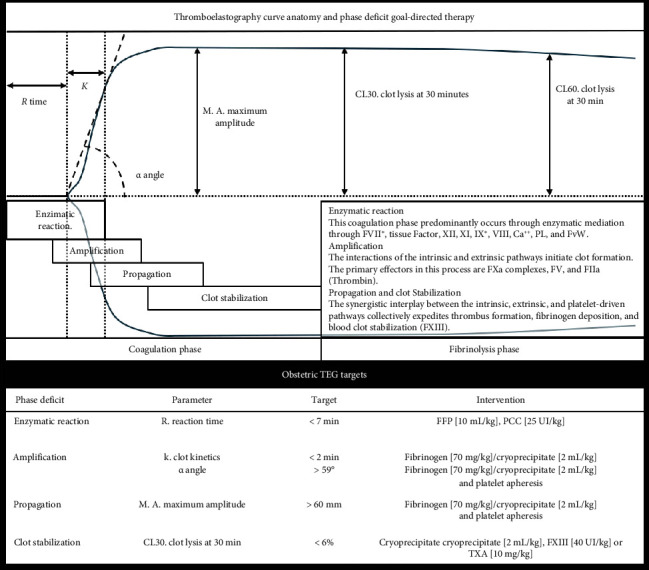
The following figure illustrates the primary sections of the thromboelastography (TEG) curve alongside their associations with the stages of the blood coagulation cascade and the predominant coagulation factors involved at each stage. A closer examination reveals that the initial phases are mediated by enzymatic activity (reaction time, *R*), followed by a latent period characterized by limited thrombin generation, moderate fibrinogen deposition, and restricted platelet activation (kinetik time, *k*). The clot formation features an accelerated rate of fibrinogen and platelet deposition (*α* angle), which coincides with enhanced platelet activation and increased enzymatic activity generated from Factor Xa complexes via the intrinsic pathway. Ultimately, clot mechanical properties stabilize because of the depletion of substrates necessary for clot formation and diminished enzymatic activity 1–3. Integrating TEG into our clinical workflow facilitates rapid decision-making within 15 min concerning the status of each phase of blood coagulation. Moreover, we integrate obstetric parameters from our study's dataset, correlating them with their respective phases. In addition, therapeutic goals and recommended hemoderivative products are carefully annotated on the accompanying visual element for each section. FFP, fresh frozen plasma; PCC, prothrombin complex concentrate; FXIII, factor XIII; TXA, tranexamic acid. (1). Shaydakov M.E., Sigmon D.F., Blebea J. Thromboelastography; 2024. https://www.ncbi.nlm.nih.gov/pubmed/23543966. (2). Hoffman M., Monroe D.M.. A cell-based model of hemostasis. Thromb Haemost. 2001; 85 (6): 958–965. doi:10.1055/s-0037-1615947. (3). Jeong Y.H., Kevin B., Ahn J.H., et al. Viscoelastic properties of clot formation and their clinical impact in East Asian versus Caucasian patients with stable coronary artery disease: a COMPARE-RACE analysis. J Thromb Thrombolysis. 2021; 51 (2):454–465. doi:10.1007/s11239-020-02240-2.

Furthermore, our center employs TEG (TEG 5000 Thrombelastograph; Haemonetics, Braintree, MA, USA) conducted by trained personnel operating the equipment, validating specimens, and interpreting curve parameters within the operating theater. This resource allows rapid and reliable coagulation assessment and decision-making abilities during active bleeding [13]. The optimization target for TEG in clinical management is a R-Time of less than 8 min; the intervention strategy is the administration of FFP at a dose of 10 mL/kg [17]. The MA goal is greater than 60 mm, and the therapeutic approach is first line with the supplemental administration of fibrinogen and adjunctive therapy of platelet apheresis. The corrective measures for an *α*-Angle above 59° are fibrinogen supplementation and platelet Apheresis based on clinical assessment [13]. A TEG schematic utility framework for evaluating each stage of the coagulation cascade, complemented by additional information on therapeutic transfusion goals, is illustrated in Figure 1.

Although our country has access to the technology, we have noticed that the principal TEG algorithms employed among our patient population primarily utilize reference intervals obtained from nonlocal sources. Beyond evaluating reference ranges, our study also explores distinct coagulation traits unique to local obstetric patients compared to their counterparts in other global regions. Our ultimate objective is to facilitate data-driven decision-making tailored to the requirements of our specialized population.

### 1.1. Research Design

This investigation is a prospective, descriptive, reference values evaluation, cross-sectional study for kaolin-based TEG parameters in previously known healthy third-trimester patients.

### 1.2. Objectives

The primary objective of this study was to establish reference values for kaolin-based TEG parameters in third-trimester pregnancy patients. The study employed high-quality control conditions in sample gathering, sampling, and TEG test processing.

## 2. Methods

### 2.1. Patient Selection

The research study received approval from the Instituto Mexicano del Seguro Social, Hospital, Unidad Médica de Alta Especialidad Hospital de Ginecología y Obstetricia, No. 23 Bioethics and Research Committee [F-2021-1905-030]. The study was performed according to the Helsinki Declaration's ethical principles and the nation's standards. We utilized the Equator Network's suggested guidelines for observational study design, specifically the Strengthening the Reporting of Observational Studies in Epidemiology (STROBE) checklist [18]. For further details, please refer to Supporting Information 1.

**Figure 2:**
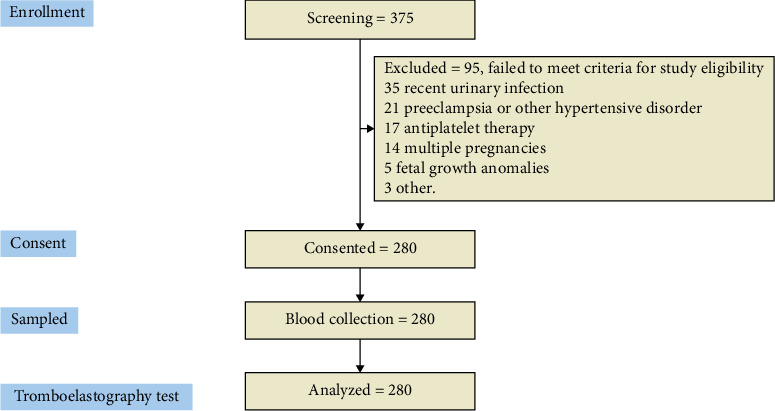
STROBE flowchart. STROBE, strengthening the reporting of observational studies in epidemiology.

The third-trimester patients included in the study were aged 18 and 38 years, recently admitted to labor, or scheduled for elective c-section. Exclusion criteria included patients with an active SARS-CoV-2 infection or those with a positive PCR test within 3 months of the previous evaluation; pregnant women with urinary tract infections, chorioamnionitis, preterm labor, multiple pregnancies, or fetal growth problems were also excluded. Pregnancy disorders such as preeclampsia, Lupus, antiphospholipid syndrome, concurrent antiplatelet therapy, signs of hepatic abnormalities, and patients with congenital or acquired coagulation disorders were also excluded. The research recruited 375 patient profiles who presented for delivery or cesarean section; however, only 280 patients met the established inclusion criteria, thus qualifying them for further analysis. Thirty-five women were excluded because they had a urinary tract infection recently. Twenty-one women were dealing with preeclampsia or other blood pressure problems that made them ineligible. Seventeen couldn't participate as they were on antiplatelet medication. Fourteen women had multiple pregnancies. Fetal development issues ruled out three expectant mothers. Five cases were eliminated for other reasons. The consort flow diagram is included in Figure 2.

### 2.2. Sample Management

Patients who participated in this study provided written consent and received a detailed gynecologic evaluation. Before being admitted to the labor or preoperative area, an OB/GYN nurse placed an 18 or 20 G intravenous catheter. Blood samples were collected for hematic cytometry, coagulation tests, and blood chemistry. To gather blood for the TEG test, the nurse used the stated IV cannula to avoid shear stress in the sample; minimize blood exposure to metal or plastic surfaces; prevent interaction with interstitial fluid, cell fragments, proteoglycans, and collagen; to avoid coagulation or fibrinolytic artifacts within the sample. The blood specimen was immediately placed in a 1 mL kaolin activator vial (TEG 5000, Hemostasis System Kaolin, Haemonetics, IL, USA) and mixed with the reagents for 40 s. Then, 0.36 mL of kaolin-activated blood was placed in a clear coagulation cup (TEG 5000, Hemostasis System disposable cups, Haemoscope Corporation, IL, USA). The TEG test on the TEG 5000 (Haemonetics, IL, USA) was conducted for 60 min at 37°C. The time since the sample collection and the start of the TEG test was below 60 s, as the hospital quality control requires. Measurements and patient data were downloaded from the software platform (TEG V4 version 4.2.101) in ⁣^∗^.txt format to ensure data reliability. The research team also gathered the patient's clinical information and blood test results from their clinical files.

### 2.3. Data Management and Statistical Analysis

The present study applied a sample size calculation method adjusted for a regression-based reference limit approach. The calculation considered a 95% confidence interval and the observation power (Z*α*/*β*), a margin of error of 10% (Δ), one-tailed variance in the parameters (Zp = 1.645), and a standard deviation (SD) of 1 in the sample distribution. The reference values were determined using the Hoffman method. This approach involves estimating the parametric cumulative frequency for a given quantitative variable and calculating a least-square linear regression over the cumulative frequency. The best-fitting equation is then used to predict the upper (*z* = 1.96) and lower (*z* = −1.96) limits for the reference intervals from the cumulative frequency vector. The study's descriptive analysis comprises the average, SD, reference values for quantitative variables, and valid total and percent for categorical variables. To provide further insight into the manuscript, the research team reviewed relevant publications on third-trimester obstetric patients, calculated the effect size using Cohen's *δ*, and reported the corresponding *p* values. All data management and statistical analyses were conducted using *R* 4.2.

## 3. Results

The analysis included the clinical records and samples from those 280 patients in their third trimester who were either admitted to labor or underwent elective c-sections. These patients had a median age of 26 (IQR 22–31) and experienced at least two previous pregnancies (IQR 1–3). The demographic profiles, blood tests, and coagulation results obtained from the samples are presented in Table 1.

The viscoelastic analysis provided several essential parameters for the patient's blood coagulation profile. Enzymatic activity was evaluated using the R-Time (*R*), which ranged between 1 and 7 min. Clot kinetics (K) were faster than the average adult, with a reference interval of 1–2  min. The rate of clot formation, measured as the angle (*α*), fell within the lower critical values, below 59 degrees of aperture. Typically, this value becomes apparent 5–8 min after clot initiation. The MA reference interval ranges from 60 to 86 mm. Clot lysis at 30 and 60 min exhibited similar values in healthy adults: 0%–6% and 0%–8%, respectively. Other important TEG parameters include curve amplitude at 30 min (A30) and amplitude at 60 min (A60); these figures closely align with the reference values derived from MA. Clot elastic strength (G) measurements in the patient's samples went from 8 to 32 dyn/cm^2^. The remaining reference intervals for the TEG parameters can be found in Table 2.

## 4. Discussion

This research provides significant evidence on the coagulation profile and reference values for pregnant women in their third trimester from the northeastern region of Mexico.

**Figure 3:**
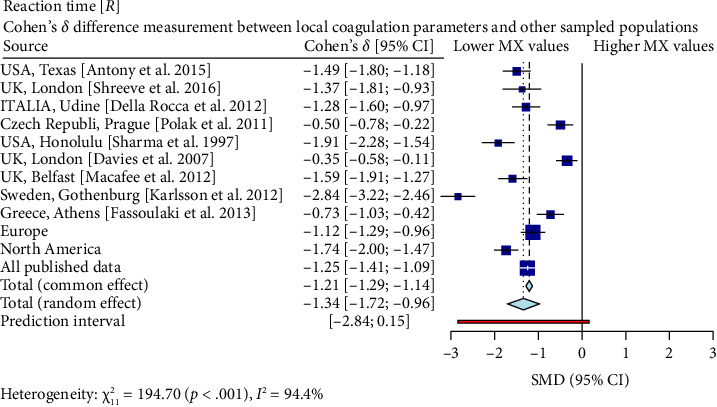
This diagram displays Cohen's *d* different measurements of coagulation reaction times between our regional sample and several international ones. Negative coefficients indicate a substantial difference between the Mexican sample and the respective study group, with the Mexican coagulation parameters exhibiting faster reaction times (except for the UK-London study by Davies, Fernando, and Hallworth). When grouping data geographically, European studies collectively present a moderate difference (*δ* = −1.12, *p* < 0.001), signifying quicker Mexican coagulation reactions. North American investigations, excluding Hawaii, yield a considerable mean Cohen's *d* score (*δ* = −1.77, *p* < 0.001), further supporting this trend. Finally, considering all available published data, the overall Cohen's *d* is −1.34, confirming faster coagulation reactions in Mexico than previously reported global findings.

**Figure 4:**
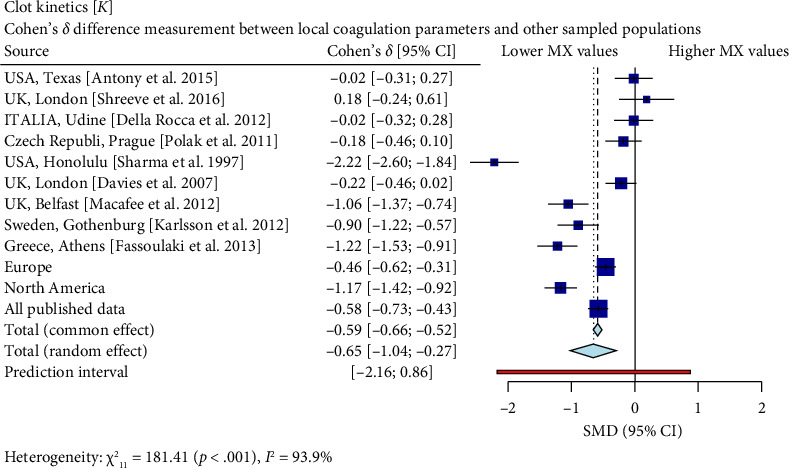
When comparing our findings with those reported in Texas, there was no significant difference between the two populations (*δ* = −0.024). However, marked disparities were observed when contrasting our samples with other geographical areas like the United Kingdom-London (*δ* = 0.22), Sweden (*δ* = −0.9), Greece (*δ* = −0.1.22), aggregated data from Europe (*δ* = −0.46), North America (*δ* = −1.17), and all published data (*δ* = −0.58). It is essential to highlight that the comparison against Sharma et al.'s sample from Honolulu yielded particularly striking results, with a noticeably significant Cohen's *δ* value of −2.22. Accordingly, these findings highlight variations among different geographical locations, which may have implications for diagnostic and therapeutic strategies in managing hemostatic abnormalities during pregnancy. It should be noted, however, that most of these comparisons show small or medium effects sizes according to Cohen's classification scheme, thus indicating only moderate deviation between groups.

**Figure 5:**
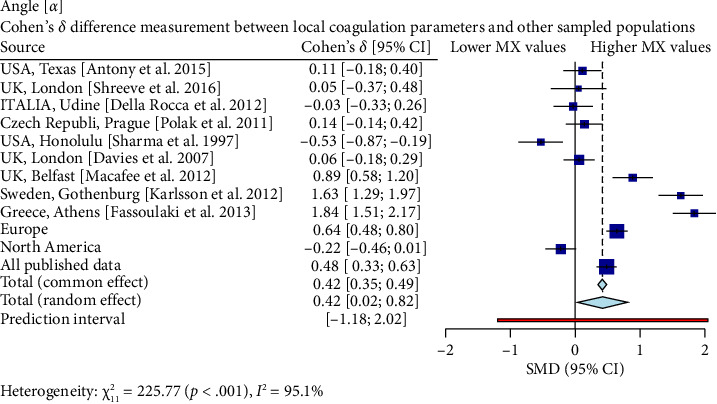
A total of 10 datasets from different geographical locations, including North America and Europe, were included in our analysis. The results indicated that there was no significant difference in the TEG curve angle of the aperture between third-trimester Mexican women and their counterparts in Texas (the United States) (*δ* = 0.11), Czech Republic (*δ* = 0.1T), or Northern America as a whole (*δ* = −0.22). However, compared to samples from Italy [20], London (the United Kingdom) [19, 23], or other European samples (*δ* = 0.64), Mexican women had significantly higher values, with large and substantial effect sizes, respectively. These findings suggest that the TEG curve angle of aperture in Mexican third-trimester pregnant women is considerably higher than previously reported in many other populations, potentially indicating differences in coagulation patterns between these groups.

**Figure 6:**
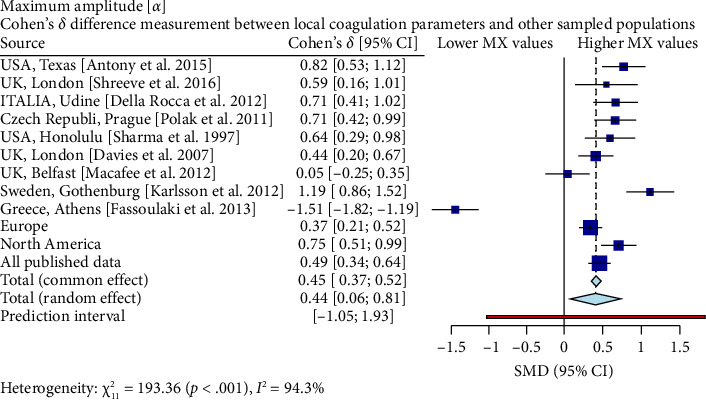
The Cohen's *d* effect sizes revealed that there was a significantly higher TEG maximum curve aperture between Mexican third-trimester women and those from the United States, Texas (*δ* = 0.82); Italy, Udine (*δ* = 0.71); Czech Republic, Prague (*δ* = 0.71); the United States, Honolulu (*δ* = 0.64); the United Kingdom, London (*δ* = 0.44); North America as a whole (*δ* = 0.75); and all published global data pooled together (*δ* = 0.46). The most prominent effects of these comparisons were noted in the contrasts involving Mexico and Sweden (*δ* = 1.19) and Mexico and Greece (*δ* = −1.51). Conversely, no substantial difference occurred in the case of the comparison between Mexico and Northern Ireland-Belfast (*δ* = 0.05).

**Figure 7:**
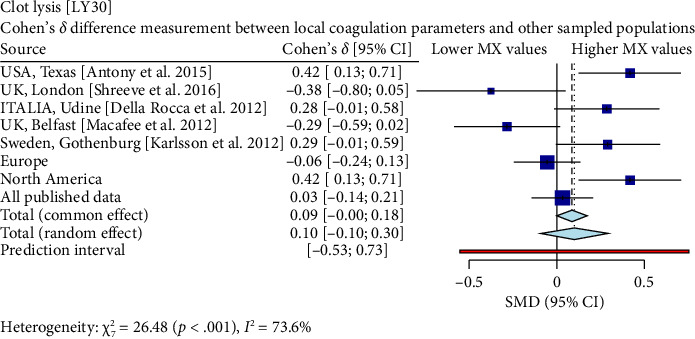
Only one site displayed a moderate effect size, which corresponds to the the United States, Texas, sample by Antony et al. (*δ* = 0.42). In contrast, a small effect size was observed at three sites: Italy; the United Kingdom, London; and Sweden, Gothenburg, where the clot lysis times among these European and Scandinavian samples slightly differed but remained comparable to the Mexican population. Two additional sites showed minor effect sizes: The United Kingdom, Belfast, sample provided by Macafee et al. had a negative value (*δ* = −0.29), implying moderately shorter clot lysis periods in Mexican participants, whereas the combined European dataset yielded negligible difference (*δ* = −0.06). Lastly, the entire collection of published data demonstrated minimal dissimilarity when assessed against the Mexican sample (*δ* = 0.032).

Interestingly, our study reveals differences between pregnant women in Mexico and those from other regions [17, 19–26]. In comparison with similar publications in terms of population and methodologies within the field, the R-Times (*R*) of our samples were significantly shorter than the published data (*δ* = −1.21, *p* < 0.001), even when compared to reports not utilizing citrate—Figure 3. Cloth kinetics (*K*) displayed minimal discrepancies with half of the published figures (the United States: Antony et al.; the United Kingdom: Shreeve et al.; ITA: Della Rocca et al.; The Czech Republic: Polak et al.; the United Kingdom.: Davies et al.). However, when categorized regionally, our patients presented shorter values compared to Europe (*δ* = −0.46, *p* < 0.001) or North America (*δ* = −1.17, *p* < 0.001)—Figure 4. Angle measurements (*α*) did not show differences in the same studies where cloth kinetics (*K*) did not. Nevertheless, our regional angle parameters differed from Europe's (*δ* = 0.64, *p* < 0.001) while being similar to North America's (*δ* = −0.22, *p*=0.062) values—Figure 5. The MA of the curve in our samples exceeded most published data, apart from the MacAfee et al. (the United Kingdom.) report (*δ* = 0.05, *p*=0.7584). Only the MA values from Fassoulaki et al. (Greece) were considerably larger than our regional amplitudes (*δ* = −1.57, *p* < 0.001). Our samples showed greater curve amplitude when compared to the European region (*δ* = 0.37, *p*=0.001) and North America (*δ* = 0.75, *p*=0.001) —Figure 6. At 30 min, cloth lysis varied regionally, displaying higher values than North American counterparts (*δ* = 0.42, *p*=0.0048); however, there were no differences against all available data (*δ* = 0.1, *p*=0.7219)—Figure 7.

Specifically, our patients demonstrated shorter R-Times, faster clotting kinetics, higher angle values, and clot amplitude compared to previously published data. These findings emphasize the importance of establishing distinct reference values for pregnant women in Mexico. As a result, we need to reassess transfusion thresholds for the use of plasma, platelets, and fibrinogen in third-trimester patients across our country or patients with Mexican backgrounds.

The changes observed with TEG mainly describe the intrinsic coagulation pathway behavior. The following areas summarize the coagulation changes in our sample: (1) increased enzymatic activity, (2) more significant platelet response, and (3) increased fibrinogen polymerization rate.

Increased enzymatic activity along the intrinsic pathway may explain the shorter R-Times observed in our patients. Variance in enzymatic activity between different populations has been reported by Cheung, Chay, and Ma who describe the diminished activity of coagulation factors II, V, VII, IX, and X among Chinese patients compared to Caucasian samples [27]. Moreover, Lutsey et al. sampled Caucasian, Hispanic, Chinese, and African-American ethnic groups and found significant differences in Factor VIII, Factor X, and thrombin activity in African-Americans [28]. Roberts et al. also found differences among the ethnic groups analyzed, with higher peak thrombin formation in African Caribbean groups [29]. The relevance of these changes in the coagulation profiles has led to new therapeutic discussions and redesign of antithrombotic therapy in Asiatic populations [30].

Platelet activity is a more intricate phenomenon that involves genetic, metabolic, and environmental factors. Under typical biological conditions, various platelet subsets have distinct aggregation rates, collagen reactivity, and biochemistry [31–33]. Nevertheless, ethnic differences in the aggregation pattern caused by the GPIIIa Pl^A1/A2^ polymorphism increased the cardiovascular risk in White South African samples when contrasted against Indian or African subjects [34]. Herrera-Galeano et al. found that changes in the platelet endothelial aggregation receptor-1 are associated with variations in cell contact reactivity; the caucasian subjects showed increased aggregation and reduced response to aspirin [35]. Moreover, Garofano et al. found 480 differentially expressed platelet-associated genes among African-American and Caucasian individuals; however, they did not assess platelet function or the impact of genomic variation [36]. The platelet genetic polymorphisms translate into platelet function variations and influence interrelationships with other blood cells. For example, Allen et al. observed that activated platelets, their association with monocytes, and their pro-thrombotic and inflammatory properties are increased in healthy African-American individuals [37].

Fibrinogen bioavailability is an essential factor in thrombus formation, and the plasma concentrations modify its interaction with thrombin, platelets, membrane, and clot stabilization by Factor XIII [38]. The plasmatic levels have been reported to contrast between ethnic groups. The MESA study found greater fibrinogen levels in Black American and Hispanic samples [28]. Jeong et al. found that East Asians had lower plasma fibrinogen levels and lower MA of the TEG than Caucasian patients [39]. Genetic polymorphism in the subunit chains may influence the synthesis rate, polymerization, or crosslinking by FXIII. Standeven et al. reported that A*α* Thr312Ala polymorphism increased fibrin diameter by facilitating FXIII's enzyme activity [40]. Other conditions, like the *β* fibrinogen gene polymorphism, are frequent in the Japanese population and have been associated with the risk of stroke [41]. Canseco et al. studied Mexican patients and found that *β* fibrinogen 455 G/A and −148 C/T polymorphisms increased plasma fibrinogen levels.

Our findings showed increased coagulation dynamics and MA, representing increased enzymatic, fibrinogen, and platelet activity. However, the biological factors determining these changes in our sample should be an area of interest for future research.

### 4.1. Specialized Technologies for Viscoelastic Coagulation Assessment

Other technologies facilitate the evaluation of the viscoelastic coagulation profile. One notable example is rotational thromboelastometry (ROTEM), which comprehensively analyzes various aspects of the coagulation cascade. Key features include the evaluation of the intrinsic (INTEM) and extrinsic coagulation pathways (EXTEM), as well as the isolation of fibrinogen polymerization dynamics (FIBTEM) [42]. ROTEM provides insights into these crucial steps, allowing for more informed decision-making regarding fibrinogen supplementation. Other tests identify the presence of heparin (HEPTEM) in the sample and fibrinolysis evaluation (APTEM) [43].

This multifaceted approach enhances the precision of transfusion therapy by allowing healthcare providers to tailor their strategies more effectively, features that TEG does not have. For instance, practitioners applying ROTEM prefer administering fibrinogen over other blood components because of its specific diagnostic indications [44].

Moreover, ROTEM accelerates the diagnosis of coagulopathy profiles by assessing curve amplitude at 5 and 10 min post-initiation [45]. The latter parameter has been identified as a predictive marker for postoperative bleeding in cardiac surgery patients. This capability significantly improves clinical outcomes by enabling timely adjustments to perioperative care [45, 46].

The application of viscoelastic assays facilitates transfusion guidance during highly complex procedures, a strategy extensively evaluated in cardiothoracic surgery. This approach reduces the utilization of blood products and highlights the significance of targeted fibrinogen administration within guided therapy protocols [47]. Notably, studies by Monaco et al. have demonstrated a decrease in adverse outcomes, including acute kidney injury, sepsis, thromboembolic complications, and allergic reactions among patients managed with the ROTEM algorithm, thereby highlighting the clinical efficacy of this tailored approach in high-complexity cardiothoracic surgery trials [45, 48, 49]. Both cardiac surgery patients and third-trimester patients sometimes face similar challenges in high-risk bleeding scenarios where timely, appropriate transfusion decisions are critical [2].

As with any analytical tool, viscoelastic assessment has inherent limitations, most notably in evaluating alterations in platelet function. Specifically, this technology detects low curve amplitude changes in patients with platelet dysfunction without showing the underlying etiology, which may be attributed to either impaired intracellular platelet mechanisms (e.g., impaired platelet responses to adenosine diphosphate) or cellular receptor abnormalities (e.g., Glanzmann Thrombosthenia) [49, 50]. Moreover, deficiencies in von Willebrand Factor will also be inadequately evaluated through this technological approach [51, 52].

### 4.2. Limitations

Although substantial evidence supports the utility of TEG technology, it is crucial to emphasize its limitations. TEG measures only the intrinsic coagulation pathway, providing insights into enzymatic activity, platelet function, and fibrinogen availability within the sampled blood. This test provides no information regarding the extrinsic coagulation pathway, congenital platelet disorders, or hypofibrinogenemia.

The study population analyzed originates from the Northern regions of Mexico. Although we believe these findings can be generalized to the broader Mexican context, caution should be exercised before directly translating them to other Latin American populations due to potential variations in demographics and healthcare practices. In addition, it is essential to highlight that our samples were not treated with citrate and were processed on-site by trained professionals. The effects of citration and transportation on blood sample stability remain unclear; however, our data suggest that untreated samples exhibited faster clotting times than those reversed from citrated status. Despite this difference, our findings still demonstrate significantly reduced R-Times compared to previous studies using noncitrated methods.

## 5. Conclusion

In the sampled third-trimester obstetric patients from the Northern region of Mexico, reference values were estimated with high precision. These patients displayed heightened coagulation activity, characterized by shorter clot R-Times, quicker clot dynamics, larger angle values, overall greater curve amplitudes, and no significant differences in enzymatic lysis activity compared to samples from other geographic regions.

### 5.1. Future Perspectives

Further studies should focus on the underlying biological (genetic, environmental, etc.) reasons behind the observed differences in coagulation dynamics. The correlation between specific genetic characteristics (e.g., GPIIIa PlA1/A2, fibrinogen, or coagulation enzymes gene polymorphisms) and coagulation patterns in pregnant women across diverse ethnic backgrounds has to be investigated.

Similar to how Asian populations' data have influenced antithrombotic therapy designs, the insights gained from studying Latin American populations could lead to more effective, tailored treatments for transfusion and antithrombotic therapy. Moreover, researchers could use machine learning algorithms with existing clinical datasets to develop predictive models capable of forecasting bleeding risks and suggesting optimal transfusion plans based on individual patient characteristics and coagulation profiles.

## Figures and Tables

**Table 1:** Sample demographics and blood test results.

Variable	Units	Median, IQR
*General characteristics*		*N* = 280
Age	years	26 [22–31]
Height	m	1.57 [1.52–1.62]
Weight	kg	68 [65–79]
Gestational age	wk	38.4 [38–40]

*Gynecologic history*		
Gestation		2 [1–3]
Parity		1 [0–2]
Abortion		0 [0–1]
C-section		1 [0–2]

*Blood test*		
Leucocytes	×1000/mm^3^	10.2 [8.3–12.8]
Hemoglobin	g/dL	11.5 [10.4–12.4]
Hematocrit	%	35.1 [31.8–37.4]
Platelet count	×1000/mm^3^	212 [175–252]
Prothrombin time	s	13 [12.3–13.8]
Activated partial thromboplastin time	s	28.5 [26.4–30.2]
Fibrinogen	g/dL	543 [444.5–639.5]
INR	—	1 [0.94–1.03]

**Table 2:** Thromboelastographic reference intervals for third-trimester obstetric patients.

Parameter	Sign	Unit	Mean, SD	Reference interval	
			*N* = 280	LL	UL
Reaction time	*R*	min	3.9, ±1.7	1	7
Clot kinetics	*K*	min	1.4, ±0.5	1	2
Angle	*α*	deg	70.4, ±5.8	59	82
Maximum amplitude	MA	mm	75.7, ±6.5	60	86
Time to maximum amplitude	TMA	min	23.4, ±4.7	14	33
Clot elastic strength	*G*	dyn/cm^2^	17.1, ±6	8	32
Clot elasticity constant	*E*	dyn/cm^2^	342.1, ±120.3	160	646
Amplitude at 30 min	A 30	mm	75.2, ±6.5	60	86
Clot lysis at 30 min	LY30	%	1.5, ±2	0	6
Amplitude at 30 min	A 60	mm	74.5, ±6.6	60	86
Clot lysis at 60 min	LY60	%	1.6, ±2.3	0	8

*Note:* Reference Interval determined with Hoffman's regression.

Abbreviations: LL, lower limit; UL, upper limit.

## Data Availability

The data that support the findings of this study are available from the corresponding author upon reasonable request. The data are not publicly available because of privacy or ethical restrictions.

## Nomenclature

TEG: Thromboelastography
GDT: Goal-directed therapy
*R*: Reaction time
*K*: Clot kinetics
*A*: Angle
MA: Maximum amplitude
TMA: Time to maximum amplitude
*G*: Clot elastic strength
*E*: Clot elasticity constant
A30: Amplitude at 30 min
LY30: Clot lysis at 30 min
A60: Amplitude at 30 min
LY60: Clot lysis at 60 min

## Supporting Information

The sequential explanation of the Strengthening the Reporting of Observational Studies in Epidemiology (STROBE) checklist has been appended as supporting information for additional guidance. We provide this document to give an overview of how we make our research adhere to the guidelines set forth by the STROBE initiative, aiming to enhance the transparency and quality of reporting in observational studies.
