# Supplementary material for: Thromboelastography Reference Values for Third-Trimester Healthy Obstetric Patients in Northern Mexico
Source: Anesthesiol Res Pract. 2025 Mar 6;2025:8871619. doi: 10.1155/anrp/8871619 (PMC11991821; doi:10.1155/anrp/8871619)
Supplement: Supporting Information — Additional supporting information can be found online in the Supporting Information section. [file 8871619.f1.docx]

Supplementary materials 1

Thromboelastography reference values for third-trimester healthy obstetric patients in northern Mexico.

**Authors**

Alvarado-Ramos, S. MD Anesthesiology ^b, c^

López-Gutiérrez, M, R. MD Anesthesiology ^a^

Nuñez-Alvar, R, D. MD Gynecology ^b^

**Affiliations**

^a^ Coordinator of Clinical Affairs, Medical Center of High Specialty Gynecology and Obstetrics No. 23. Mexican Social Security Institute. Monterrey, Nuevo León, México.

^b^ Physician, Medical Center of High Specialty Gynecology and Obstetrics No. 23. Mexican Social Security Institute. Monterrey, Nuevo León, México.

^c^ Professor, Technological Institute and Higher Studies of Monterrey. School of Medicine and Health Sciences. Monterrey, Nuevo León, México.

STROBE Statement checklist

|  | Item No | Recommendation | Page No |
| --- | --- | --- | --- |
| **Title and abstract** | 1 | Thromboelastography reference values for third-trimester healthy obstetric patients in northern Mexico. |  |
|  |  | (*b*) The abstract includes an informative and balanced summary of the manuscript in 250 words. |  |
| Introduction | | | |
| Background/rationale | 2 | The principal thromboelastography (TEG) algorithms employed among our patient population primarily utilize reference intervals obtained from non-local sources. To effectively distinguish between healthy hemostatic states and altered ones using thromboelastography (TEG)-generated targets, one must establish reference intervals carefully, tailored to specific demographics. Doing so ensures accurate discrimination while minimizing the risk of misinterpretation or inappropriate responses. |  |
| Objectives | 3 | The primary objective of this study was to establish reference values for kaolin-based thromboelastography parameters in third-trimester pregnancy patients. The study employed high-quality control conditions in sample gathering, sampling, and TEG test processing. |  |
| Methods | | | |
| Study design | 4 | This investigation is a prospective, descriptive, reference values evaluation, cross-sectional study for kaolin-based thromboelastography parameters in previously known healthy third-trimester patients. |  |
| Setting | 5 | The research study was conducted in northern Mexico in a tertiary-level health center that provides obstetrical care. |  |
| Participants | 6 | **Eligibility criteria**  third-trimester patients included in the study were aged 18 and 38 years, recently admitted to labor, or scheduled for elective c-section  **Exclusion criteria**  Exclusion criteria included patients with an active Sars CoV 2 infection or those with a positive PCR test within three months of the previous evaluation; pregnant women with urinary tract infections, chorioamnionitis, preterm labor, multiple pregnancies, or fetal growth problems were also excluded. Pregnancy disorders such as pre-eclampsia, Lupus, anti-phospholipid syndrome, concurrent antiplatelet therapy, signs of hepatic abnormalities, and patients with congenital or acquired coagulation disorders were also excluded. |  |
|  |  |  |  |
| Variables | 7 | Demographic data, blood test results, and thromboelastography measurements |  |
| Data sources/ measurement | 8* | **Thromboelastography**  The samples were processed by trained personnel. The blood reagent was Kaolin (TEG® 5000, Hemostasis System Kaolin, Haemonetics, IL, USA) mixed for 40 seconds. Then, 0.36 mL of kaolin-activated blood was placed in a clear coagulation cup (TEG® 5000, Hemostasis System disposable cups, Haemoscope Corporation, IL, USA). The thromboelastography test on the TEG 5000 (Haemonetics, IL, USA) was conducted for 60 minutes at 37°C. Measurements and patient data were downloaded from the software platform (TEG® V4 version 4.2.101) in *.txt format to ensure data reliability. |  |
| Bias | 9 | Our Research implemented the following approach to address bias  **Samples**  Employing a standardized protocol for blood sample collection (using an 18/20 G intravenous catheter before admission to labor/preoperative areas, minimizing contact with potential contaminants, and immediate mixing with reagents).  **TEG process**  The personnel entrusted with the operation of the TEG underwent an extensive training program, ensuring their proficiency in its management.  Utilizing the Hoffman method for determining reference values based on cumulative frequency and least-squares linear regression provides a systematic approach to establishing normative ranges, potentially reducing biases inherent in more straightforward estimation techniques.  Accounting for factors like confidence interval, observational power, margin of error, and variance when calculating the sample size aims to ensure the dataset is sufficiently powered, thereby reducing type II errors and associated biases. |  |
| Study size | 10 | The study applied a sample size calculation method adjusted for a regression-based reference limit approach. The calculation considered a 95% confidence interval and the observation power (Zα/β), a margin of error of 10% (Δ), one-tailed variance in the parameters (Zp = 1.645), and a standard deviation of 1 (D) in the sample distribution. |  |
| Quantitative variables | 11 | Descriptive Statistics: For each quantitative thromboelastography parameter, the study reports the average (Mean) To give a central tendency measure of what a typical value might look like among the participants. Standard Deviation (SD): To quantify each parameter's variation or dispersion around its mean. |  |
| Statistical methods | 12 | (*a*) Describe all statistical methods.  Reference Values Determination: Method Used: The Hoffman method was chosen for determining reference values. This approach involves estimating the parametric cumulative frequency for each quantitative variable. Calculating a least-squares linear regression over the cumulative frequencies to find the best-fitting equation.  Groupings Chosen Based on Reference Intervals: Upper Limit Calculation: z = +1.96 to define the upper boundary of the normal range for each parameter. Lower Limit Calculation: z = -1.96 to define the lower boundary of the normal range for each parameter. These specific z-values are chosen because they correspond to approximately 95% confidence levels for establishing reference ranges when assuming a Gaussian distribution of the parameters. By doing so, about 5% of healthy individuals' measurements will fall outside this range due to random variability rather than actual health issues related to the parameters measured. |  |
|  |  | (*b*) Describe any methods used to examine subgroups and interactions  Comparative Insights via Effect Size Calculation: Cohen's δ: Used to calculate the standardized difference between two means. P-Values Reporting: Accompanying the effect sizes to indicate whether observed differences could be attributed to chance at a particular significance level (commonly set at α=0.05). |  |
|  |  | (*c*) Explain how missing data were addressed  Records with incomplete data capture were excluded from the calculations |  |
|  |  |  |  |
|  |  |  |  |
| Results | | |  |
| Participants | 13* | (a) Report numbers of individuals at each stage of study  Numbers Potentially Eligible: 375 The total number of patient profiles initially considered for screening.  Examined for Eligibility: Same as above since all were assessed against set criteria. All 375 underwent initial screening based on predefined inclusion criteria.  Confirmed Eligible: 280 patients whose profiles matched the inclusion criteria after initial assessment.  Included in the Study (Received Intervention/Assessment): 280 All eligible participants ' data was utilized since the study focused on analyzing existing clinical records and biological samples without introducing new interventions.  Completing Follow-Up: Not Applicable (N/A). Given that the study analyzed pre-existing medical records and did not introduce any longitudinal component requiring follow-up visits, this category does not apply here.  Analyzed: 280 All confirmed eligible participants' data was subjected to thorough analysis as part of the study's methodology. |  |
|  |  | (b) Give reasons for non-participation at each stage |  |
|  |  | (c) Consider use of a flow diagram  The study includes one |  |
| Descriptive data | 14* | (a) Give characteristics of study participants (eg demographic, clinical, social) and information on exposures and potential confounders  Characteristics of Study Participants:  Demographic Characteristics: Median Age: 26 years.Interquartile Range (IQR): 22-31 years. Number of Previous Pregnancies: At least 2 (with an IQR of 1-3). Clinical Characteristics: Pregnancy Trimester: Third trimester. Reason for Admission/Care: Delivery or Caesarean Section (C-section)  Potential Confounders:  The research focuses secondary on the comparison of thromboelastography (TEG) parameters across different regions and against existing literature, without explicit mention of specific lifestyle, medical treatment, or environmental exposures affecting the outcomes:  Geographical Location: Regional comparisons suggest potential confounding due to genetic, dietary, healthcare system differences, etc., between regions (Europe, North America).  Sample Collection Methodology: Using citrate vs. non-citrate methods might affect some TEG parameter readings but seems controlled for comparative analyses.  These items are addressed in the study limitations section |  |
|  |  |  |  |
|  |  |  |  |
| Outcome data | 15* | Report numbers of outcome events or summary measures over time |  |

| Main results | 16 | (*a*) Give unadjusted estimates and, if applicable, confounder-adjusted estimates and their precision (eg, 95% confidence interval). Make clear which confounders were adjusted for and why they were included  Confounder-Adjusted Estimates  There is limited information provided that directly supports adjusting for confounders beyond what could be inferred from the discussion about regional variations in outcomes. Thus, without explicit mention of how potential confounding variables (e.g., age, number of previous pregnancies, elective C-section vs. labor admission) might influence the analyses differently across regions, providing adjusted estimates based solely on the given text isn't feasible. |  |
| --- | --- | --- | --- |
|  |  | (*b*) Report category boundaries when continuous variables were categorized  Not Applicable |  |
|  |  |  |  |
| Other analyses | 17 | Report other analyses done—eg analyses of subgroups and interactions, and sensitivity analyses  This study undertakes supplementary analyses comparing the viscoelastic measurements reported in its publications with those from other populations, highlighting key differences observed between these datasets. Furthermore, the limitations encountered during this comparative analysis are also presented. |  |
| Discussion | | | |
| Key results | 18 | Summarise key results with reference to study objectives In the sampled third-trimester obstetric patients from the Northern region of Mexico, reference values were estimated with high precision. These patients displayed heightened coagulation activity, characterized by shorter clot reaction times, quicker clot dynamics, larger angle values, overall greater curve amplitudes, and no significant differences in enzymatic lysis activity compared to samples from other geographic regions. |  |
| Limitations | 19 | Discuss limitations of the study  Although substantial evidence supports the utility of Thromboelastography (TEG) technology, it is crucial to emphasize its limitations. TEG measures only the intrinsic coagulation pathway, providing insights into enzymatic activity, platelet function, and fibrinogen availability within the sampled blood. This test provides no information regarding the extrinsic coagulation pathway, congenital platelet disorders, or hypofibrinogenemia.  The study population analyzed originates from the northern regions of Mexico. Although we believe these findings can be generalized to the broader Mexican context, caution should be exercised before directly translating them to other Latin American populations due to potential variations in demographics and healthcare practices. Additionally, it is essential to highlight that our samples were not treated with citrate and were processed on-site by trained professionals. The effects of citration and transportation on blood sample stability remain unclear; however, our data suggest that untreated samples exhibited faster clotting times than those reversed from citrated status. Despite this difference, our findings still demonstrate significantly reduced reaction times compared to previous studies using non-citrated methods. |  |
| Interpretation | 20 | Give a cautious overall interpretation of results considering objectives, limitations, multiplicity of analyses, results from similar studies, and other relevant evidence  Overall Interpretation  This study investigates the viscoelastic analysis (TEG) of coagulation profiles in pregnant women during their third trimester in the northeastern region of Mexico. The main objective was establishing reference values for this specific population, considering previous studies indicating regional and ethnic disparities.  Key Findings: Third-trimester healthy patients from Mexico have Shorter reaction times, faster clotting kinetics, higher angle values, and increased maximum amplitude. These results differ significantly from existing literature on other geographic and ethnic cohorts, underscoring the necessity for tailored, location-specific guidelines for transfusion strategies in third-trimester pregnancies within Mexico or in patients of Mexican descent. |  |
| Generalisability | 21 | Discuss the generalisability (external validity) of the study results  While this study provides valuable insights into the coagulation profile and reference values for pregnant women in their third trimester within the northeastern region of Mexico, several limitations affect the generalisability of the results:  Geographic and Ethnically Limited Sample Pool: The study was conducted exclusively in the northeastern region of Mexico, which might limit the applicability of the findings to other parts of Mexico or globally. Differences in lifestyle, diet, genetics, and healthcare systems could lead to varying coagulation profiles.  Single-Trimester Focus: Concentrating solely on the third trimester leaves a gap in knowledge regarding how coagulative parameters evolve across gestational periods. Longitudinal studies could offer valuable data for personalized prenatal care plans.  7Genetic and Environmental Factors: Although correlations with enzymatic activity, platelet response, and fibrinogen polymerization rate are noted, deeper investigation into underlying genetic predispositions, lifestyle factors, or environmental influences contributing to these enhanced coagulatory states remains unexplored, leaving room for detailed mechanistic studies. |  |
| Other information | | | |
| Funding | 22 | Give the source of funding and the role of the funders for the present study and, if applicable, for the original study on which the present article is based  This study was supported by internal funding from Instituto Mexicano del Seguro Social, and no external financial or material supports were provided. The funders had no role in the study design, data collection, analysis, interpretation, writing of the report, or decision to submit the paper for publication |  |

*Give information separately for exposed and unexposed groups.

**Note:** An Explanation and Elaboration article discusses each checklist item and gives methodological background and published examples of transparent reporting. The STROBE checklist is best used in conjunction with this article (freely available on the Web sites of PLoS Medicine at http://www.plosmedicine.org/, Annals of Internal Medicine at http://www.annals.org/, and Epidemiology at http://www.epidem.com/). Information on the STROBE Initiative is available at http://www.strobe-statement.org.
